# Cell adhesion is regulated by CDK1 during the cell cycle

**DOI:** 10.1083/jcb.201802088

**Published:** 2018-09-03

**Authors:** Matthew C. Jones, Janet A. Askari, Jonathan D. Humphries, Martin J. Humphries

**Affiliations:** Wellcome Trust Centre for Cell-Matrix Research, Faculty of Biology, Medicine and Health, Manchester Academic Health Science Centre, University of Manchester, Manchester, England, UK

## Abstract

In most tissues, anchorage-dependent growth and cell cycle progression are dependent on cells engaging extracellular matrices (ECMs) via integrin–receptor adhesion complexes. In a highly conserved manner, cells disassemble adhesion complexes, round up, and retract from their surroundings before division, suggestive of a primordial link between the cell cycle machinery and the regulation of cell adhesion to the ECM. In this study, we demonstrate that cyclin-dependent kinase 1 (CDK1) mediates this link. CDK1, in complex with cyclin A2, promotes adhesion complex and actin cytoskeleton organization during interphase and mediates a large increase in adhesion complex area as cells transition from G1 into S. Adhesion complex area decreases in G2, and disassembly occurs several hours before mitosis. This loss requires elevated cyclin B1 levels and is caused by inhibitory phosphorylation of CDK1–cyclin complexes. The inactivation of CDK1 is therefore the trigger that initiates remodeling of adhesion complexes and the actin cytoskeleton in preparation for rapid entry into mitosis.

## Abstract (precis)

Adherent cells round up before dividing, but how this is linked to the cell cycle is unclear. Jones et al. demonstrate that CDK1 promotes adhesion complex formation and increases cell adhesion area from G1 to S phase. Subsequently, inhibitory phosphorylation of CDK1 by cyclin B1 triggers adhesion complex disassembly during G2 in preparation for mitosis.

## Introduction

The cell cycle is a tightly regulated process that orchestrates genome duplication and accurate distribution of DNA and other factors into daughter cells after mitosis. Progression through the cell cycle is primarily mediated by members of the cyclin-dependent kinase (CDK) family in association with partner cyclin proteins (Malumbres, 2014), with entry into mitosis being controlled by the activation of the cyclin B–CDK1 complex (also known as mitosis promoting factor; Lohka et al., 1988; Labbe et al., 1989; Gautier et al., 1990). Activity of cyclin B1–CDK1 is tightly regulated via several feedback loops (Lindqvist et al., 2009), and during G2, inactive cyclin B1–CDK1 is maintained in the cytosol after phosphorylation of CDK1 at Y15 by Wee1 and related kinases to prevent premature entry into mitosis (Gould and Nurse, 1989; Parker and Piwnica-Worms, 1992). The activity of cyclin B1–CDK1 increases progressively once cells enter prophase (Gavet and Pines, 2010b), and active cyclin B1–CDK1 translocates to the nucleus (Gavet and Pines, 2010a), triggering several mitotic events such as cell rounding, nuclear envelope breakdown, chromosome condensation, and spindle formation.

For most cells, cell cycle progression is anchorage-dependent (Fang et al., 1996; Schulze et al., 1996), requiring cell–ECM interactions via integrin transmembrane receptors and the formation of actin-associated adhesion complexes (Zhu et al., 1996; Renshaw et al., 1997; Roovers et al., 1999; Mettouchi et al., 2001; Welsh et al., 2001; Park et al., 2011). Before entry into mitosis, adhesion complexes are rapidly disassembled, and cells retract from their surroundings and round up to divide (Cramer and Mitchison, 1997; Yamakita et al., 1999; Maddox and Burridge, 2003; Dao et al., 2009). This cell rounding is required for accurate spindle formation and chromosome capture (Carreno et al., 2008; Kunda et al., 2008; Kunda and Baum, 2009; Lancaster et al., 2013). Furthermore, integrin-mediated adhesion is required for determining the orientation of cell division (Théry et al., 2005) and for efficient cytokinesis to occur (Aszodi et al., 2003; Reverte et al., 2006; Pellinen et al., 2008; Högnäs et al., 2012; Mathew et al., 2014). However, the molecular mechanism that couples the cell cycle machinery to the regulation of cell adhesion via integrin-associated adhesion complexes is unknown.

In this study, we demonstrate that the regulation of adhesion complexes and remodeling of the actin cytoskeleton occurs in a cell cycle–dependent manner. As cells transitioned from G1 to S, we observed a CDK1-dependent increase in adhesion complex area mediated in part via phosphorylation of the formin FMNL2. Upon entry into G2, adhesion complex area decreased, and actin became more peripherally distributed. The loss of adhesion complexes in G2 was mediated by increased cyclin B1 levels and subsequent inhibition of CDK1 by Wee1. Remodeling of adhesion complexes was required for cells to subsequently round up and undergo efficient mitosis because preventing the changes resulted in an increase in failed mitoses and multinucleation. Collectively, these data demonstrate that CDK1 inhibition is the trigger that initiates adhesion remodeling in preparation for entry into mitosis and reveal an intimate link between the cell cycle machinery and cell–ECM adhesion.

## Results

### Adhesion complexes are modified in a cell cycle–dependent manner

**Figure 1.**
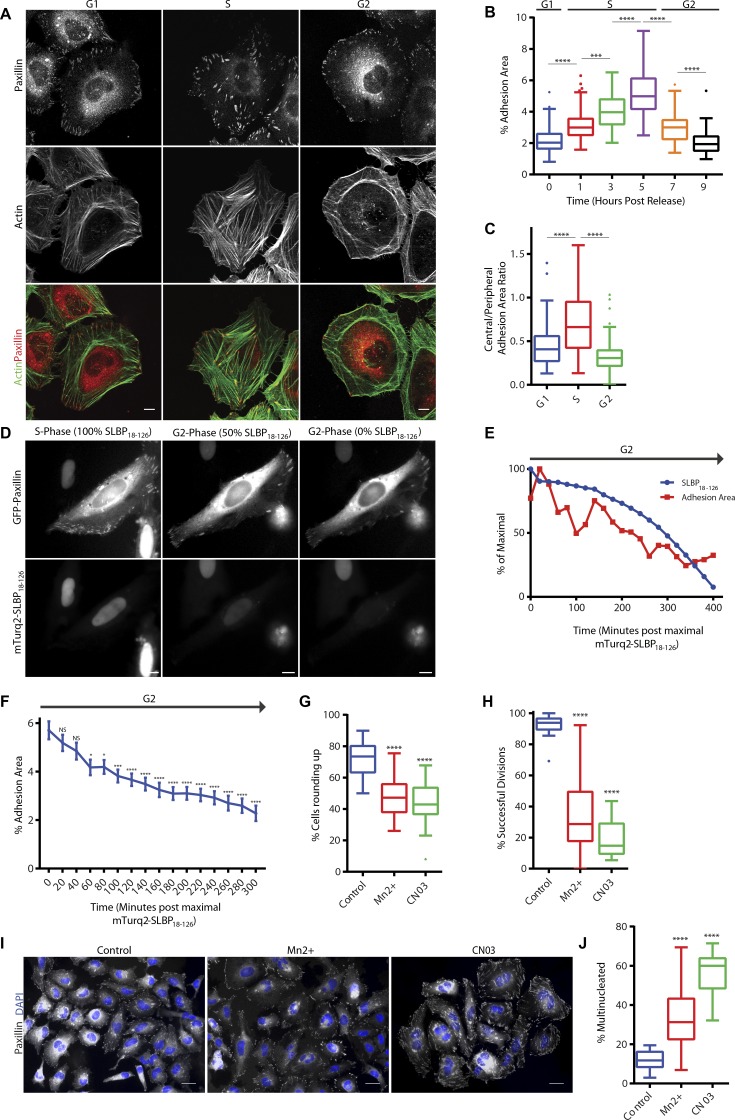
**Adhesion complex area is modified in a cell cycle–dependent manner. (A)** Immunofluorescence images of cells in G1, S, and G2 phase stained for adhesion marker paxillin and actin. **(B)** Quantification of adhesion complex area per cell over a period of 0- to 9-h release after double-thymidine block. A minimum of 61 cells per condition was used for analysis. **(C)** Quantification of ratio of central adhesion complex area (adhesion complex area >3 µm from cell periphery) to peripheral adhesion complex area (adhesion complex area <3 µm from cell periphery) of cells in G1, S, and G2 phase. A minimum of 56 cells per condition was used for analysis. **(D)** Fluorescence images of a HeLa cell expressing mTurq2-SLBP_18-126_ and GFP-paxillin illustrating progressive loss of mTurq2-SLBP_18-126_. **(E)** Quantification of GFP-paxillin adhesion area and mTurq2-SLBP_18-126_ intensity changes over a time period of 400 min for an individual cell illustrating loss of mTurq2-SLBP_18-126_ and associated decrease in adhesion area. **(F)** Quantification of GFP-paxillin adhesion complex area in cells after degradation of mTurq2-SLBP_18–126_ and progression through G2. A total of 30 cells was used for analysis. **(G and H)** Quantification of a HeLa cell rounding and successful division after Mn^2+^ or CN03 treatment. A minimum of 2,483 cells per condition was used for analysis. **(I)** Immunofluorescence images of synchronized HeLa cells treated with Mn^2+^ or CN03 that have progressed through a single division stained for paxillin and with DAPI. Bars: (A and D) 10 µm; (I) 20 µm. **(J)** Quantification of HeLa cell multinucleation after treatment with Mn^2+^ or CN03. A minimum of 384 cells per condition was used for analysis. Results in B, C, G, H, and J are displayed as Tukey box and whisker plots (whiskers represent 1.5× interquartile range) and are for at least three biological replicates. *, P < 0.05; ***, P < 0.001; ****, P < 0.0001.

Initially, we performed a detailed characterization of the changes in adhesion complex architecture that take place through the cell cycle. For this purpose, HeLa cells were synchronized by double-thymidine block, released from the block for various time points reflecting presence in G1, S, and G2 (Fig. S1, A and B), and fixed and stained for paxillin (as a marker of adhesion complexes) and F-actin. Consistent with S as a period of cell growth, the adhesion complex area per cell increased from G1 to S (Fig. 1, A and B; and Fig. S1 C). The pattern of adhesion complexes also changed from a predominantly peripheral location in G1 to sites that were distributed throughout the cell body in S (Fig. 1, A and C; and Fig. S1 C). On entry into G2, the adhesion complex area decreased (Fig. 1, A and B; and Fig. S1 C), and the distribution reverted to the peripheral pattern observed in G1 (Fig. 1, A and C; and Fig. S1 C). The actin cytoskeleton was modified concomitantly with the observed changes in adhesion complexes: in G1 and G2, F-actin was found predominantly at the cell periphery, whereas cells in S exhibited centrally spanning stress fibers (Figs. 1 A and S1 C). Similar observations were also made when vinculin was used as an alternative marker of adhesion complexes (Fig. S1 D) and in synchronized U2OS cells (Fig. S1 E). Furthermore, the decrease in adhesion complex area observed in G2 was confirmed in live-cell analysis of cells coexpressing GFP-paxillin and mTurq2-stem–loop binding protein 18–126 (SLBP_18–126_). Degradation of mTurq2-SLBP_18–126_ occurs at the end of S (Bajar et al., 2016) and can therefore be used as a marker for the entry into G2. Consistent with observations made in fixed cells, adhesion complex area decreased progressively as cells progressed through G2 (Fig. 1, D–F; and Video 1). These observations therefore define cell cycle–dependent modification of adhesion complexes and the cytoskeleton and highlight the typical remodeling that takes place in preparation for entering mitosis. However, rather than adhesion complex disassembly taking place immediately upon the onset of mitosis as is currently assumed from the rapid rounding that occurs at M, these data indicate that an initial remodeling of adhesion complexes and the cytoskeleton is actually triggered earlier, during G2.

To determine the functional relevance of cell cycle–dependent adhesion remodeling in G2, either actin stress fiber or adhesion complex disassembly in G2 was suppressed by treatment of G1 cells with a RhoA activator (CN03), which prevents RhoA-GTP hydrolysis and locks RhoA in an active state, or manganese, which hyperactivates integrins (Fig. S1 E; Mould et al., 1995, 2002). Both of these approaches inhibited cell rounding (Fig. 1 G and Videos 2, 3, and 4), without suppressing cell cycle progression into G2 (Fig. S1 F), and greatly diminished the ability of those cells that did round to undergo successful cell division (Fig. 1 H and Videos 2, 3, and 4). Both treatments also elicited an increase in the number of multinucleated daughter cells (Fig. 1, I and J). These data demonstrate that the controlled modification of either cell–ECM adhesion or the actin cytoskeleton in G2 is essential to allow cells to pass through mitosis accurately and that, consistent with previous observations (Dao et al., 2009; Lancaster et al., 2013; Marchesi et al., 2014), manipulations that prevent adhesion complex disassembly before mitosis reduce the ability of cells to divide efficiently.

### CDK1 kinase activity is required to maintain adhesion complexes in interphase

**Figure 2.**
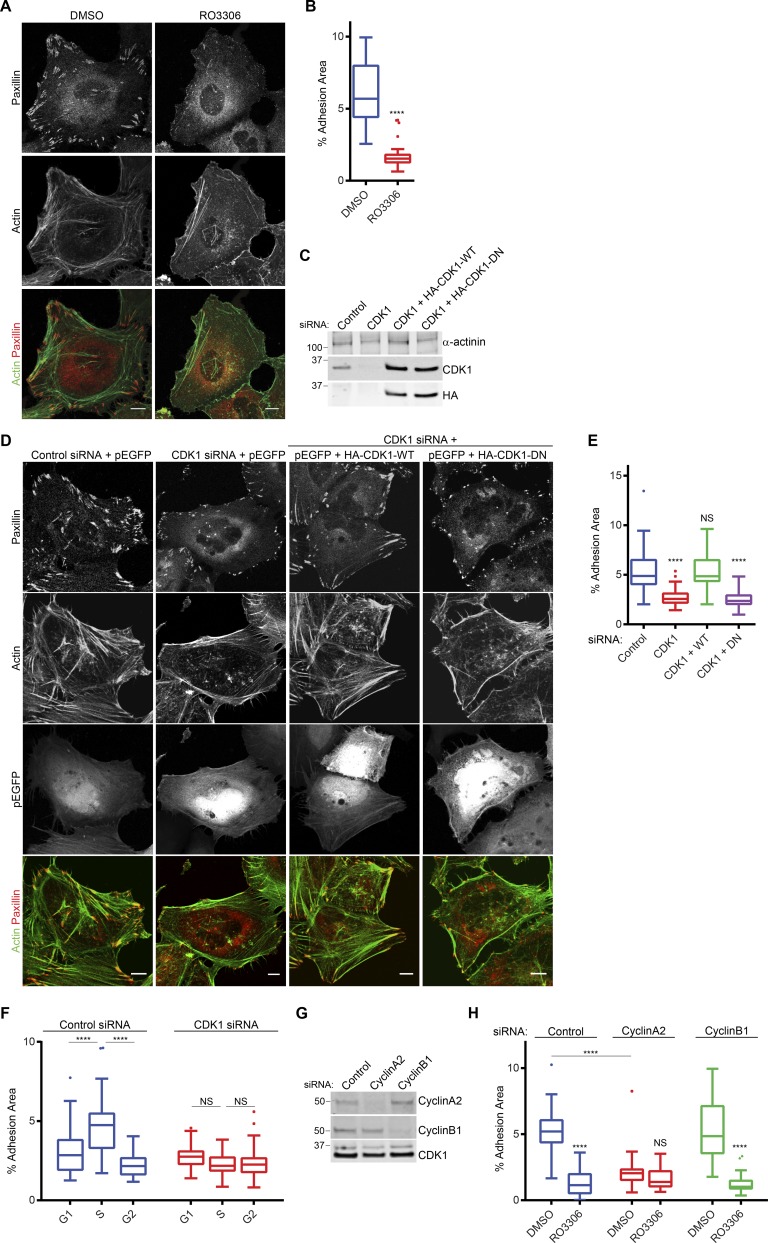
**CDK1 kinase activity maintains adhesion complexes. (A)** Immunofluorescence images of cells plated on glass coverslips for 48 h and then treated with either DMSO or CDK1 inhibitor RO3306 for 1 h and stained for paxillin and actin. **(B)** Quantification of adhesion complex area per cell of DMSO or RO3306 treated cells. A minimum of 32 cells per condition was used for analysis. **(C)** Western blot showing knockdown of endogenous CDK1 and expression of HA-tagged CDK1. **(D)** Immunofluorescence images of GFP-positive control, CDK1-knockdown cells, and CDK1-knockdown cells reexpressing WT or dominant-negative CDK1 stained for paxillin and actin. **(E)** Quantification of adhesion area per cell after CDK1 knockdown and reexpression. A minimum of 48 cells per condition was used for analysis. Bars, 10 µm. **(F)** Quantification of changes in adhesion area per cell in G1, S, and G2 phase for control and CDK1-knockdown cells. A minimum of 36 cells per condition was used for analysis. **(G)** Western blot showing knockdown of endogenous cyclin A2 and cyclin B1. Molecular masses are given in kilodaltons. **(H)** Quantification of adhesion complex area per cell in control, cyclin A2–, or cyclin B1–knockdown cells treated with DMSO or RO3306. A minimum of 36 cells per condition was used for analysis. Results in B, E, F, and H are displayed as Tukey box and whisker plots (whiskers represent 1.5× interquartile range) and are for at least three biological replicates. *, P < 0.05; ****, P < 0.0001.

CDK1 is a promiscuous serine/threonine kinase that can phosphorylate hundreds of proteins, with a great number of these being involved in regulation of cellular architecture (Olsen et al., 2010; Petrone et al., 2016). Based on phosphoproteomic analyses, adhesion complexes contain a range of potential mitotic kinase phosphorylation sites, raising the possibility that these enzymes might regulate adhesion directly (Robertson et al., 2015). Consistent with this hypothesis, we reported previously that inhibition of CDK1 results in a loss of actin stress fibers and the formation of small nascent adhesion complexes at the cell periphery (Robertson et al., 2015). Because a loss of adhesion complexes and actin stress fibers is characteristic of cells in G2, we hypothesized that CDK1-dependent regulation of adhesion complexes may be central to the changes observed during cell cycle progression. Inhibition of CDK1 kinase activity in asynchronous HeLa cells with three different compounds reduced the adhesion complex area per cell and induced a loss of stress fibers (Fig. 2, A and B; and Fig. S2 E). In contrast, treatment with inhibitors targeting CDK2 or CDK4/6, which reduced cell viability after long-term treatment (Fig. S2 D) or induced G1/S arrest in synchronized cells (Fig. S2 C), had no effect on adhesion complexes or the actin cytoskeleton (Fig. S2, A and B). RNAi-mediated knockdown of CDK1 also decreased the adhesion complex area in a manner that was rescued by reexpression of WT CDK1 but not dominant-negative kinase-dead CDK1 (Fig. 2, C–E; van den Heuvel and Harlow, 1993). Together, these data demonstrate a specific role for CDK1 kinase activity in maintaining integrin adhesion complexes. Furthermore, the changes in the adhesion complex area and distribution observed during the cell cycle (Fig. 1) were lost in synchronized cells after CDK1 knockdown (Figs. 2 F and S2, F–H), demonstrating that cell cycle–dependent changes in adhesion complexes before mitosis are dependent on CDK1 and identifying a novel nonmitotic role for CDK1 in regulating adhesion complexes and the actin cytoskeleton.

CDK1 activity is predominantly mediated by interaction with cyclins A2 and B1 (Gong and Ferrell, 2010); therefore, the cyclin-binding partner mediating CDK1-dependent regulation of adhesion complexes was assessed by RNAi knockdown of either cyclin A2 or cyclin B1 (Fig. 2 G). In an asynchronous population, knockdown of either cyclin A2 or cyclin B1 did not result in arrest of cells in G2 (Fig. S2 I); however, knockdown of cyclin A2 resulted in a significant decrease in the adhesion complex area and a peripheral distribution of adhesion complexes and actin reminiscent of CDK1 inhibition or cells in G2, whereas knockdown of cyclin B1 had no effect (Figs. 2 H and S2 J). Furthermore, treatment of cyclin A2 knockdown cells with CDK1 inhibitor did not further reduce the adhesion complex area, whereas a significant decrease was observed in cyclin B1–knockdown cells (Figs. 2 H and S2 J). These data demonstrate that the role of CDK1 in regulating adhesion complexes and the cytoskeleton during interphase is likely to be dependent on its binding to cyclin A2 rather than cyclin B1.

**Figure 3.**
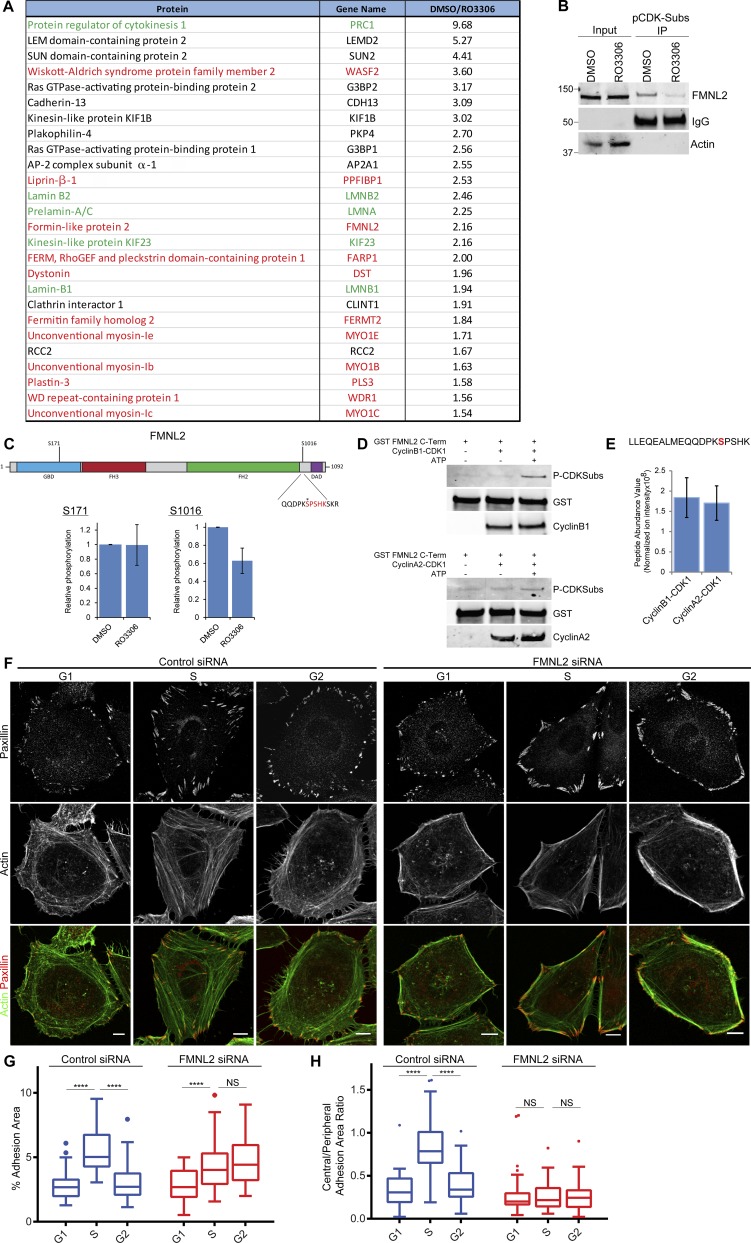
**FMNL2 is a CDK1 substrate required for cell cycle–dependent modification of adhesion complexes. (A)** MS identification of CDK1 substrates showing CDK1-dependent phosphorylated proteins from interphase cells. Proteins highlighted in green are known CDK1 substrates, and those in red are linked to regulation of adhesion complexes and actin. **(B)** Immunoprecipitation (IP) of anti-CDK/MAPK substrate antibody and Western blot of FMNL2 from DMSO- or RO3306-treated cells. **(C)** Schematic diagram of FMNL2 phosphorylation sites found by quantitative MS after treatment with RO3306. Asterisk indicates serine1016 site of phosphorylation. **(D)** Western blots showing phosphorylation of a C-terminal fragment of FMNL2 by purified cyclin A2–CDK1 or cyclin B1–CDK1. Molecular masses are given in kilodaltons. **(E)** MS identification of a single phosphopeptide corresponding with S1016 after in vitro phosphorylation of a C-terminal fragment of FMNL2 by purified cyclin A2–CDK1 or cyclin B1–CDK1. **(F)** Immunofluorescence images of control or FMNL2-knockdown cells in G1, S, and G2 phase stained for adhesion marker paxillin and actin. Bars, 10 µm. **(G)** Quantification of adhesion area changes in G1, S, and G2 phase for control and FMNL2-knockdown cells. A minimum of 45 cells per condition was used for analysis. **(H)** Quantification of ratio of central adhesion complex area (adhesion complex area >3 µm from cell periphery) to peripheral adhesion complex area (adhesion area <3 µm from cell periphery) in G1, S, and G2 phase for control and FMNL2-knockdown cells. A minimum of 40 cells per condition was used for analysis. Results in C and E are displayed as bar graphs ± SEM and in G and H as Tukey box and whisker plots (whiskers represent 1.5× interquartile range) and are for at least three biological replicates. ****, P < 0.0001.

CDK1 signaling has been linked to changes in the actin cytoskeleton during mitosis and cytokinesis via regulation of Rho guanine nucleotide exchange factors (RhoGEFs) such as Ect2, GEF-H1, and leukemia-associated RhoGEF (LARG; Birkenfeld et al., 2007; Matthews et al., 2012; Helms et al., 2016) as well as other actin-associated proteins such as filamins, WDR1, and formins (Cukier et al., 2007; Kuilman et al., 2015; Ramanathan et al., 2015). To identify potential novel targets of CDK1 in nonmitotic cells, a mass spectrometry (MS)-based approach was used to identify proteins in nonsynchronized cells that were phosphorylated on CDK substrate motifs in a CDK1-dependent manner. Immunoprecipitation with an anti-CDK/MAPK substrate motif antibody identified 26 proteins whose abundance decreased by ≥1.5-fold after CDK1 kinase inhibition (Fig. 3 A). Among these proteins were many known CDK1 substrates (Fig. 3 A, green) and several actin-associated proteins (Fig. 3 A, red), highlighting that CDK1 is able to phosphorylate multiple proteins involved in cytoskeletal regulation. Of particular interest was the formin FMNL2 because this has previously been linked to stimulation of actin fiber formation downstream of RhoC activation (Kitzing et al., 2010; Zeng et al., 2015). FMNL2 has a single predicted consensus CDK1 phosphosite at S1016 (High Stringency, Scansite 3.0; Obenauer et al., 2003), and CDK1-dependent phosphorylation of FMNL2 was confirmed by Western blotting after CDK substrate immunoprecipitation (Fig. 3 B). Furthermore, MS-based phosphosite mapping of affinity-purified mCherry-FMNL2 detected phosphorylation of FMLN2 on residues S171 and S1016, and phosphorylation at S1016 was reduced after CDK1 inhibition in asynchronous cells (Fig. 3 C). Purified cyclin A2–CDK1 and cyclin B1–CDK1 were both able to phosphorylate a GST-tagged C-terminal fragment of FMNL2 directly (Fig. 3 D), and this phosphorylation was confirmed to occur at S1016 by MS-based phosphosite mapping (Fig. 3 E). No significant difference in abundance of S1016 phosphorylated peptide was observed between GST-FMNL2 C terminus incubated with cyclin A2–CDK1 or cyclin B1–CDK1 (Fig. 3 E), demonstrating that CDK1 associated with either cyclin A2 or cyclin B1 is capable of phosphorylating FMNL2 at S1016 in vitro.

To test the functional role of CDK1-dependent FMNL2 phosphorylation, a phosphomimetic FMNL2-S1016E mutant was expressed in CDK1-knockdown cells. In contrast with expression of WT FMNL2, expression of FMNL2-S1016E partially rescued the loss of adhesion complex area observed in asynchronous cells after CDK1-knockdown (Fig. S3 A), demonstrating that phosphorylation of FMNL2 at S1016 contributes to the regulation of adhesion complexes downstream of CDK1. These data together with the MS data presented in this study (Fig. 3 A) and in previous research (Robertson et al., 2015) also suggest that in addition to FMNL2, CDK1 is potentially able to regulate multiple proteins that control adhesion complexes and the actin cytoskeleton.

We next sought to determine the role of FMNL2 in the CDK1-dependent modification of adhesion complexes during the cell cycle. In synchronized cells, Western blotting after CDK substrate immunoprecipitation demonstrated similar levels of FMNL2 phosphorylation in G1 and S followed by a reduction in FMNL2 phosphorylation in G2 (Fig. S3 C), suggesting that increased FMNL2 phosphorylation did not contribute to adhesion complex growth in S phase but that dephosphorylation may facilitate adhesion complex disassembly in G2. Consistent with this, FMNL2 knockdown or expression of nonphosphorylatable FMNL2-S1016A resulted in loss of stress fibers and predominantly peripheral adhesion complexes in G1 and S (Figs. 3 F and S3 D). During S, these peripheral adhesion complexes increased in area (Fig. 3, F and G; and Fig. S3, B and D), but no associated change in central adhesion complexes as seen in control cells was observed (Figs. 3 H and S3 C). Furthermore, the reduction in adhesion complexes after transition from S to G2 did not occur after knockdown of FMNL2 or expression of FMNL2-S1016A (Fig. 3, F and G; and Fig. S3, B and D). CDK1-dependent phosphorylation of FMNL2 at S1016 is therefore required for the formation of central adhesion complexes and centrally spanning actin stress fibers with dephosphorylation in G2 contributing to adhesion complex and stress fiber disassembly. In the absence of CDK1-dependent phosphorylation of FMNL2 at S1016, peripheral adhesion complexes increased in area during S (Figs. 3 F and S3, B and D), suggesting that stimulation of the adhesion complex area in S phase occurs via FMNL2-independent pathways but were not subsequently disassembled in G2. Together, these data demonstrate that FMNL2 is a novel interphase substrate of CDK1 involved in the maintenance of transcellular stress fibers and adhesion complexes during S and the facilitation of cell cycle–dependent changes in adhesion after modulation of CDK1 activity and subsequent decrease in FMNL2 S1016 phosphorylation.

### Cyclin B1 levels regulate CDK1 activity to control adhesion complex disassembly in G2

**Figure 4.**
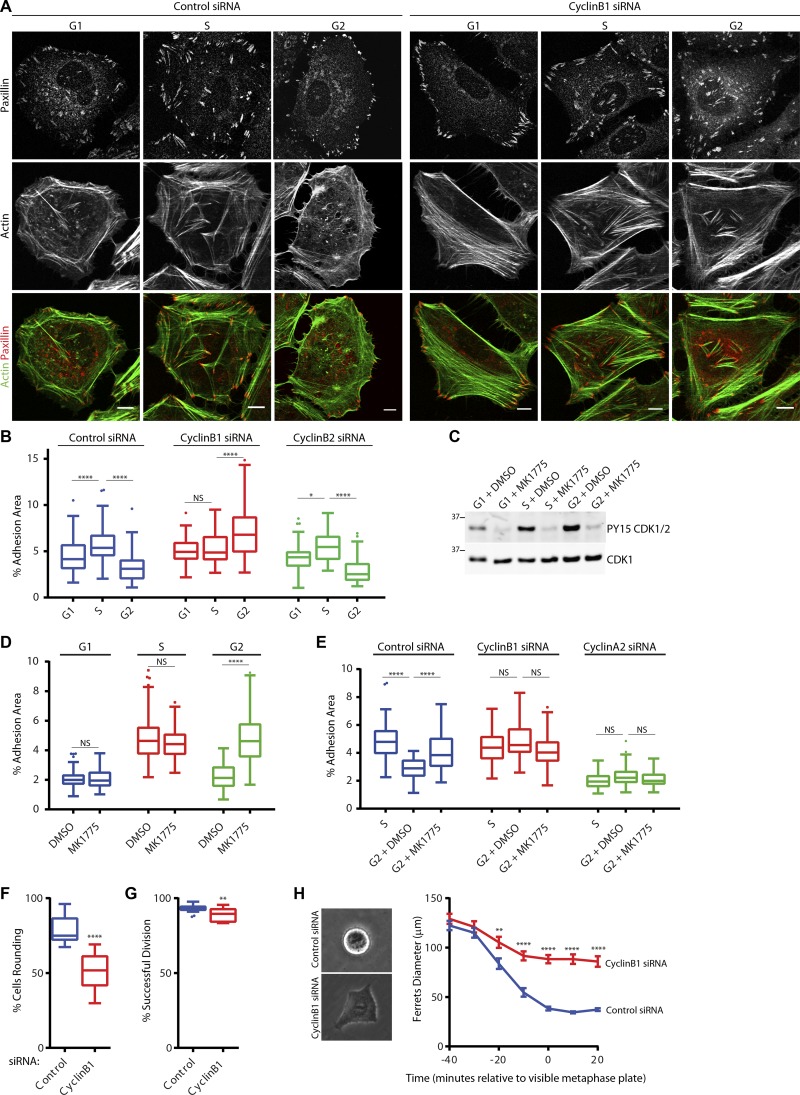
**Increased cyclin B1 levels mediate adhesion disassembly in G2 phase. (A and B)** Immunofluorescence images of control or cyclin B1–knockdown cells stained for paxillin and actin (A), and quantification of adhesion complex area changes in G1, S, or G2 phase for control, cyclin B1–, and cyclin B2–knockdown cells (B). A minimum of 43 cells per condition was used for analysis. Bars, 10 µm. **(C)** Western blot of CDK1-Y15 phosphorylation for Wee1 inhibitor (MK1775)-treated cells in G1, S, and G2, and quantification of adhesion area changes across the cell cycle with cells in G1, S, and G2 being treated with either DMSO or MK1775 for 2 h. Molecular masses are given in kilodaltons. **(D)** Quantification of adhesion area changes in G1, S, and G2 cells being treated with either DMSO or MK1775 for 2 h. **(E)** Quantification of adhesion area changes in G2 cells being treated with either DMSO or MK1775 for 2 h after knockdown of either cyclin A2 or cyclin B1. **(F and G)** Quantification of HeLa cell rounding and successful division for control and cyclin B1–knockdown cells. A minimum of 2,348 cells per condition was used for analysis. **(H)** Quantification of HeLa cell Ferret’s diameter as cells enter mitosis in control (36 cells) and cyclin B1–knockdown cells (35 cells). Results in B and D–G are displayed as Tukey box and whisker plots (whiskers represent 1.5× interquartile range) and are for at least three biological replicates. *, P < 0.05; **, P < 0.01; ****, P < 0.0001.

Regulation of CDK1 activity during the cell cycle is primarily mediated by complexing with the cyclins A2 and B1 (Gong and Ferrell, 2010). The key molecular event associated with G2 is increased cyclin B1 expression where it associates with CDK1 in preparation for entry into mitosis. During G2, the activity of cyclin B1–CDK1 is tightly inhibited by phosphorylation of CDK1 at Y15 by Wee1 to prevent premature entry into mitosis (Gould and Nurse, 1989; Parker and Piwnica-Worms, 1992). We hypothesized therefore that the reduction in adhesion complexes observed during G2 might be coordinated with the induction of cyclin B1 expression and the subsequent inactivation of CDK1. Indeed, a higher proportion of CDK1 was associated with both cyclin A2 and cyclin B1 in G2 relative to that observed in S (Fig. S4, A and B). The levels of tyrosine-15–phosphorylated CDK1 associated with each cyclin were also increased in G2 (Fig. S4, A and B), and CDK1, cyclin B1, cyclin A2, and Wee1 were all observed in the cytosolic fraction of cells in G2 (Fig. S4 C), demonstrating that G2 is associated with an inactivation of CDK1 in the cytosol after increased association with cyclin B1 and cyclin A2 and inhibitory phosphorylation by Wee1. RNAi-mediated knockdown of cyclin B1, but not the closely related cyclin B2, abrogated the ability of cells to disassemble adhesion complexes in G2 (Fig. 4, A and B; and Fig. S4, E–H), suggesting that increased levels of cyclin B1 are required to inactivate CDK1 and promote adhesion complex disassembly in G2.

To determine whether loss of adhesion complexes in G2 was a consequence of the accumulation of Wee1-dependent inhibition of CDK1 activity, short-term treatment of cells in G1, S, and G2 with MK1775, a Wee1 inhibitor that reduces CDK1 tyrosine-15 phosphorylation (Fig. 4 C) and thus hyperactivates CDK1–cyclin complexes (Gheghiani et al., 2017), was used. Hyperactivation of CDK1–cyclin complexes in G2 resulted in an increase in the adhesion complex area (Fig. 4 C), whereas treatment of cells in G1 and S elicited no change (Fig. 4 C). This suggests that the reduction of the adhesion complex area observed in G2 is a consequence of the formation of inactive CDK1–cyclin complexes in this phase. Consistent with observations made in asynchronous cells (Fig. 2 H), knockdown of cyclin A2 resulted in a reduction in the adhesion complex area in S cells, suggesting a role for cyclin A2–CDK1 in promoting adhesion complex formation (Fig. 4 E) during this cell cycle phase. Furthermore, hyperactivation of cyclin B1–CDK1 alone (after RNAi knockdown of cyclin A2) was unable to promote adhesion complex formation in G2 (Fig. 4 E), indicating that cyclin B1–CDK1 is unable to regulate adhesion complexes in the same manner as cyclin A2–CDK1 and that the two complexes have opposing effects on adhesion complex area. These data therefore demonstrate that the key event that occurs in G2 to trigger loss of adhesion complexes and thereby prime cells for rapid entry into mitosis is the increased expression levels of cyclin B1. Indeed, altering the levels of cyclin B1 in cells via the overexpression of nondegradable cyclin B1 (Clute and Pines, 1999) either in asynchronous cells or in cells synchronized in S resulted in a loss of adhesion complexes similar to that seen in G2 (Fig. S4, I–L), demonstrating the key role for cyclin B1 levels in facilitating adhesion complex disassembly. Furthermore, treatment of asynchronous cells expressing nondegradable cyclin B1 with MK1775 rescued the observed decrease in adhesion complex formation, without having an effect on control cells (Fig. S4 J), demonstrating that the reduction in the adhesion complex area associated with overexpression of cyclin B1 occurs as a result of increased Wee1-dependent phosphorylation and inactivation of CDK1. Consistent with a key role for cyclin B1 in regulating mitotic entry (Jackman et al., 2003; Krämer et al., 2004; Soni et al., 2008; Gavet and Pines, 2010a,b; Gong and Ferrell, 2010), knockdown of cyclin B1 resulted in a significant decrease in the number of cells rounding up to divide (Fig. 4 F) together with a small but significant decrease in the ability of mitotic cells to undergo successful division (Fig. 4 G). During mitosis, cells undergo a major shape change characterized by cell rounding and the formation of a rigid cortical actin network (Stewart et al., 2011; Ramanathan et al., 2015). This shape change is mediated by several factors including increased Ect2-driven RhoA activity (Matthews et al., 2012), Rap1 inhibition (Dao et al., 2009), adhesion complex disassembly (Dao et al., 2009), and increased osmotic pressure (Stewart et al., 2011). After cyclin B1 knockdown, mitotic cells were unable to round up to the same degree as control cells (Fig. 4 H), demonstrating a key role for cyclin B1 in mediating the morphological changes that occur during mitotic cell rounding.

**Figure 5.**
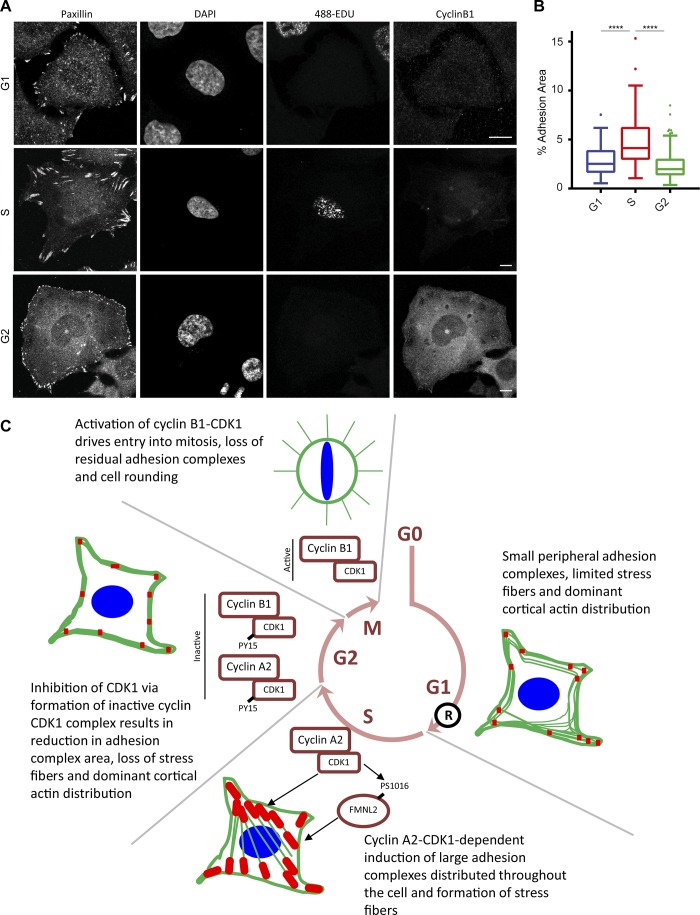
**Adhesion area in asynchronous cells varies depending on cell cycle phase. (A)** Immunofluorescence images of asynchronous cells pulse-labeled with EDU and then stained for paxillin and cyclin B1. G1 cells were defined as EDU and cyclin B1 negative, S cells were EDU positive, and G2 cells were cyclin B1 positive. Bars, 10 µm. **(B)** Quantification of adhesion complexes in asynchronous cells segregated into G1, S, and G2 as defined in A. A minimum of 106 cells per condition was used for analysis. **(C)** Schematic diagram of model presented in this paper. Cellular focal adhesion area changes as cells progress through the cell cycle, with an initial cyclin A2–CDK1-dependent increase in adhesion area and stress fibers in S phase being observed. In G2, inhibition of CDK1 after association with cyclin B1 and phosphorylation by Wee1 results in a reduction in focal adhesion area and stress fibers. Residual focal adhesions are subsequently disassembled after activation of cyclin B1–CDK1, entry into mitosis, and mitotic cell rounding. Results in B are displayed as Tukey box and whisker plots (whiskers represent 1.5× interquartile range) and are for at least three biological replicates. ****, P < 0.0001.

To further explore the role of cyclin B1 levels in promoting reduction in the adhesion complex area, asynchronous cells were pulse labeled with 5-ethylene-2-deoxyuridine (EDU) for 30 min to identify cells in S and fixed and stained for cyclin B1 and paxillin (Fig. 5 A), and then the adhesion complex area quantified. This approach allowed identification of cells in G1 (EDU and cyclin B1 negative), S (EDU positive and cyclin B1 negative), and G2 (cyclin B1 positive) and demonstrated that consistent with synchronized cells (Fig. 1, A and B) and cells overexpressing GFP-paxillin and mTurq2-SLBP (Fig. 1, D–F), the adhesion complex area was increased in EDU-positive S cells and that cyclin B1–positive G2 cells had a subsequently reduced adhesion complex area (Fig. 5 B). Therefore, cyclin B1 levels can be used within an asynchronous population to identify cells in G2 and as an indicator of cells with a reduced adhesion complex area.

## Discussion

In summary, our major findings identify an intimate association between the cell cycle machinery and cell adhesion by defining (A) cell cycle–dependent changes in adhesion complex area, (B) a nonmitotic role for CDK1 in regulating cell–matrix adhesion complexes in part via phosphorylation of the formin FMNL2, and (C) a mechanism by which this activity can be switched off in a cell cycle–coordinated fashion via the increased expression of cyclin B1 in G2. These insights therefore explain how regulating the activity of CDK1 results in modification of adhesion complexes and the cytoskeleton as cells progress through the cell cycle.

The regulation of adhesion complex composition and turnover has to date largely focused on migrating cells or cells spreading onto matrix proteins. In this study, we have described a novel aspect of adhesion complex regulation that centers on cell cycle progression. Cell adhesion complex area increases as cells progress from G1 into S and subsequently decreases as cells enter G2. These changes are supported by recent observations that cellular contractile forces follow the same trend through the cell cycle (Vianay et al., 2018) and therefore suggest a concerted cell cycle–dependent regulation of adhesion.

A central regulator of this process is CDK1 because perturbation of CDK1 results in a loss of cell cycle–dependent adhesion changes. CDK1 substrates include regulators of the actin (Yamashiro et al., 1991, 2001; Kuilman et al., 2015; Ramanathan et al., 2015), intermediate filament (Chou et al., 1990; Yamaguchi et al., 2005) and tubulin networks (Andersen et al., 1997; Liakopoulos et al., 2003) together with regulators of Rho GTPases (Birkenfeld et al., 2007; Matthews et al., 2012; Whalley et al., 2015; Helms et al., 2016). Regulation of these pathways may allow CDK1 to facilitate the significant changes in cell morphology and reuse of cytoskeletal polymers that are required for mitosis to occur, but in large part they have been linked to regulation of adhesion complexes during interphase. A previous phosphoproteomic analysis of adhesion complex components identified a large number of potential CDK substrates (Robertson et al., 2015), suggesting a fundamental role for CDK1 in regulating adhesion complexes in interphase via multiple pathways that have yet to be elucidated. This role is distinct from the induction of mitosis associated with cyclin B–CDK1 because knockdown of cyclin B1 did not have an impact upon adhesion complex formation. The data presented in this study suggest that cyclin A2 is required for CDK1-dependent regulation of adhesion complexes in interphase; therefore, additional cyclin A2–CDK1-specific substrates involved in regulating adhesion complexes may exist. In the future, it would be instructive to determine whether additional regulators of CDK1 activity such as the CKS proteins (Krishnan et al., 2010) or novel binding partners of CDK1 act to promote its activity and function in interphase.

Modification of CDK1 activity may therefore not only influence the proliferative potential of cells but could also have an impact upon other adhesion-dependent processes such as cell migration and invasion. In support of this, CDK1 activity has recently been shown to function downstream of the protein tyrosine phosphatase LAR to regulate adhesion complex formation in mouse embryonic fibroblasts stimulated with platelet-derived growth factor (Sarhan et al., 2016), and expression of CDK1 is required for αvβ3 integrin–dependent stimulation of prostate cancer cell migration (Manes et al., 2003). Therefore, further investigation of this novel nonmitotic role for CDK1 in regulating adhesion complexes and the cytoskeleton is required.

Mitotic cell rounding allows accurate spindle positioning and chromosome separation (Carreno et al., 2008; Kunda et al., 2008; Kunda and Baum, 2009; Lancaster et al., 2013). Consistent with previous observations, we have demonstrated that prevention of adhesion complex disassembly perturbs mitosis (Dao et al., 2009; Lancaster et al., 2013; Marchesi et al., 2014). Several proteins that regulate adhesion complexes are also required for efficient cell division; for example, the Rho-GEFs LARG, and GEF-H1 mediate mechanical force on adhesion complexes (Guilluy et al., 2011) but are also required for efficient mitosis (Birkenfeld et al., 2007; Helms et al., 2016) and activation of Rho, and the promotion of myosin-dependent contractility are required for both mitotic cell rounding and cytokinesis to occur (Maddox and Burridge, 2003; Glotzer, 2005; Matthews et al., 2012; Breznau et al., 2015). Furthermore, formin activity is required to maintain cortical actin in mitotic cells (Ramanathan et al., 2015). It is logical, therefore, that this regulatory machinery needs to be recycled and redistributed away from promoting adhesion complexes and actin stress fibers for reuse during cell division. Our findings show that disassembly of adhesion complexes and modification of the cytoskeleton begins in G2, before cell retraction and rounding up. This is manifested in the formation of peripheral adhesion complexes and a switch to a more cortical actin distribution and prepares the cell for the rapid rounding up required once the G2/M checkpoint has been passed. These findings suggest that modulation of adhesion complexes and the cytoskeleton represents a key process that occurs in G2 in preparation for efficient mitosis. Because perturbation of G2-dependent adhesion complex disassembly results in a loss of accurate cell division, we speculate that this may help explain how changes in the tissue microenvironment that influence the actin cytoskeleton and adhesion complex formation and signaling such as the elevated ECM stiffness that characterizes many carcinomas (Faurobert et al., 2015) contribute to aneuploidy and tumor progression.

In this study we have identified the formin FMNL2 as a novel substrate for CDK1 that plays a role in maintaining adhesion complexes and facilitates cell cycle–dependent changes in adhesion complexes. Knockdown of FMNL2 or expression of a nonphosphorylatable S1016A mutant resulted in the loss of adhesion complexes and stress fibers within the cell body, with peripheral structures being maintained. This is consistent with a role for FMNL2 in promoting elongation of Arp2/3-branched actin networks (Block et al., 2012) and transcellular stress fiber formation (Péladeau et al., 2016). FMNL2 has previously been shown to accumulate at the edge of lamellipodia and at the tips of filopodia in migrating B16-melanoma cells (Block et al., 2012; Kage et al., 2017), where in conjunction with FMNL3, it regulates actin filament formation throughout the protruding lamellipodia (Block et al., 2012; Kage et al., 2017). How FMNL2 influences the formation of adhesion complexes, however, remains to be determined. FMNL2 may play a direct role in the formation of adhesion complex–associated stress fibers; alternatively, it may provide the transcellular framework from which dorsal and ventral stress fibers along with focal adhesions subsequently form (Hotulainen and Lappalainen, 2006). This alternative is consistent with the observation that adhesion complex growth can occur in the absence of FMNL2 during S phase, suggesting that FMNL2 is not directly responsible for adhesion complex growth in S phase, but in its absence, the localization of adhesion complexes alters because of changes in the existing actin network. How FMNL2 mediates actin dynamics during cell cycle progression and how this influences adhesion complex formation will form the basis of future studies. Furthermore, how phosphorylation of FMNL2 at S1016 modulates its activity and the role for this phosphorylation in regulating actin dynamics during cell cycle progression and migration remains to be determined. Phosphorylation of FMNL2 at S1072 by PKC has previously been shown to be required for translocation of FMNL2 in association with β1 integrin from the plasma membrane to intracellular vesicles (Wang et al., 2015). Therefore, cross talk between these phosphorylation sites may take place that regulate integrin trafficking and subsequently adhesion complex localization and turnover. FMNL2 phosphorylation at S1016 has been identified in large-scale phosphoproteomic analyses of both mitotic and interphase cells (Dephoure et al., 2008; Olsen et al., 2010). Alongside our observation that purified cyclin B1–CDK1 is able to phosphorylate FMNL2 S1016, this suggests that FMNL2 may also be phosphorylated in mitosis by cyclin B1–CDK1. Given that formins have been linked to the maintenance of cortical actin in mitotic cells (Ramanathan et al., 2015) and cytokinesis (Bohnert et al., 2013), a possible mitotic role for FMNL2 warrants further investigation and potentially provides a good example of an additional protein that plays a dual role in regulating adhesion complexes during interphase and the cytoskeleton during mitosis.

Cyclin B1 levels and the amount of CDK1 associated with both cyclin B1 and cyclin A2 increased as cells entered G2 (Figs. S1 and S4, A and B). During G2, cyclin–CDK1 complexes are maintained in an inactivate state via phosphorylation at Y15 by Wee1 (Gould and Nurse, 1989; Parker and Piwnica-Worms, 1992). This accumulation of inactive cyclin–CDK1 complexes provides a simple means of reducing the complex adhesion maintenance activity of CDK1 at a stroke and acts as a temporal switch to trigger loss of adhesion complexes coordinated with entry into G2. The key event that drives loss of adhesion complexes in G2 is the increase in cyclin B1 levels and this, alongside the recent observations that the cytoplasmic localization of inactive cyclin B1–CDK1 determines the length of G2 and is required to prevent premature entry into mitosis (Strauss et al., 2018), demonstrates a role for cytoplasmic inactive cyclin B1–CDK1 in coordinating cellular changes in G2. Although a great deal is known regarding the signaling events facilitating activation of cyclin B1–CDK1 and entry into mitosis, very little is known about the signaling events that mark the transition from S to G2. This warrants further work as several questions remain. For example, how does increased cyclin B1 influence Wee1 or Myt1 activity, and how is expression, activity, or subcellular localization of the myriad of other cell cycle and cytoskeletal regulators determined by cyclin B1?

## Materials and methods

### Cell culture and synchronization

HeLa and U2OS cells (European Collection of Cell Cultures 93021013 and 92022711; Sigma-Aldrich) were maintained in DMEM (Sigma-Aldrich) supplemented with 10% (vol/vol) FCS (Lonza), 1% (vol/vol) penicillin/streptomycin, and 2 mM l-glutamine at 37°C, 5% (vol/vol) CO_2_. For steady-state analysis of adhesion complexes in asynchronous cells, cells were cultured on glass coverslips for 48 h and then treated with indicated compounds for 1 h. HeLa and U2OS cells were synchronized by using a double-thymidine block protocol. Cells were plated and after 24 h of growth, thymidine was added to a final concentration of 2 mM, and the cells were incubated for 16 h. Cells were then washed twice with PBS and allowed to grow for 8 h in fresh DMEM. Thymidine was then added to a final concentration of 2 mM for an additional 16 h before cells were washed twice with PBS and released into DMEM.

### Cell cycle analysis

HeLa and U2OS cells were arrested in G1/S by double-thymidine block, and at the indicated times after release, cells were either fixed for immunofluorescence analysis or flow cytometry. For flow cytometric analysis, cells were trypsinized and fixed with 50% (vol/vol) ethanol then washed with PBS and stained with 50 µg/ml propidium iodide and treated with 50 µg/ml RNaseA for 1 h at RT. Samples of 10,000 cells were then analyzed by using BD LSR Fortessa. For live-cell microscopy of dividing cells, cells were plated onto 6- or 12-well tissue culture dishes as appropriate then grown at 37°C and 5% (vol/vol) CO_2_. Cells were arrested in G1 by double-thymidine block then released into complete medium. Where appropriate, cells were treated with 400 µM Mn^2+^ or 1 mg/ml CN03 RhoA activator, and brightfield images were acquired subsequently on an AS MDW live-cell imaging system (Leica Biosystems) by a 10× Plan Apochromat glycerine objective. Point visiting using Image Pro 6.3 (Media Cybernetics) allowed multiple positions to be imaged within the same time course, and cells were maintained at 37°C and 5% (vol/vol) CO_2_. The images were collected at 10-min intervals by using a Cascade II electron-multiplying charge-coupled device camera (Photometrics). For live-cell analysis of adhesion modification in G2, HeLa cells stably expressing mTurq2-SLBP_18–126_ were plated on glass-bottom 24-well tissue culture dishes and then subsequently transiently transfected with GFP-paxillin. Fluorescent images of cells undergoing division were acquired on an Eclipse Ti inverted microscope (Nikon) by using a 20×/0.45 SPlan Fluor objective using imaging software NIS Elements (AR.46.00.0; Nikon). Point visiting was used to allow multiple positions to be imaged within the same time course, and cells were maintained at 37°C and 5% CO_2_. The images were collected by using a Retiga R6 (Q-Imaging) camera, and GFP-positive cells that were observed to round up and undergo successful mitosis during the time period of the video were subsequently used to analyze adhesion changes in G2. The proportion of dividing cells, successful divisions, and adhesion area per image were quantified by using ImageJ (National Institutes of Health).

### Immunofluorescence microscopy

HeLa or U2OS cells were fixed in 4% wt/vol PFA for 15 min, washed twice with PBS, and permeabilized by using 0.2% wt/vol Triton X-100 in PBS for 5 min. Cells were then washed with PBS and PFA quenched by incubation with 0.1 M glycine/PBS for 15 min. Cell were washed with PBS three times and then incubated with primary antibodies (45 min at RT) and washed with PBS containing 0.1% wt/vol Tween-20 (PBST) and incubated for 30 min with the appropriate secondary antibodies and, where applicable, Alexa Fluor dye–conjugated phalloidin (Thermo Fisher Scientific). Finally, cells were washed three times with PBST and once with distilled H_2_O before being mounting on coverslips by using ProLong diamond antifade reagent (Thermo Fisher Scientific) and imaging.

Images were acquired on an inverted confocal microscope (TCS SP5 Acousto-Optical Beam Splitter; Leica Microsystems) by using a 63× objective (HCX Plan Apochromat, NA 1.25) and Leica Confocal Software (Leica Microsystems), and image analysis was performed using ImageJ. Images were background subtracted by using rolling ball subtraction, and images of paxillin staining were thresholded to define adhesion complexes. By using a size cut-off of 0.2 µm, the total area of paxillin-positive adhesion complexes was determined per cell as a proportion of total cell area. Representative cells were selected based on consistency with phenotype observed across the field of view, with distinct cells present within a similar density of surrounding cells being chosen for analysis.

### Reagents

Monoclonal antibodies used were mouse anti–FAK (clone 77, 1:1,000; 610088; BD), rabbit anti–FAK pY397 (clone 141-9, 1:1,000; 44–625G; Invitrogen), mouse anti–paxillin (clone 349, 1:10,000 for immunoblotting, 1:300 for immunofluorescence; 610051; BD), mouse anti–cyclin B1 (clone GNS3, 1:2,000; 05–373; EMD Millipore), mouse anti–cyclin A2 (clone BF683, 1:1,000; 4656; Cell Signaling Technology), mouse anti–CDK1 (clone POH1, 1:1,000; 4656; Cell Signaling Technology), mouse anti-FMNL2 (1:1,000; ab57963; Abcam), mouse antivinculin (clone hVin-1, 1:2,000 for immunoblotting, 1:300 for immunofluorescence; V9264; Sigma-Aldrich), mouse antiactin (clone AC-40, 1:2,000; A3853; Sigma-Aldrich), mouse anti–α-actinin (clone BM-75.2, 1:1,000; A5044; Sigma-Aldrich), mouse anti-HA (clone 12CA5, 1:2,000; MA1-12429 Thermo Fisher Scientific), rabbit anti-CDK1 pY15 (clone 10A11, 1:1,000; 4539; Cell Signaling Technology), and rabbit anti–Rb pS807/811 (clone D20B12, 1:1,000; 8516; Cell Signaling Technology). Polyclonal antibodies used were rabbit anti-CDK1 (1:1,000; ABE1403; EMD Millipore), rabbit antipaxillin (pY118; 1:1,000; 44-722G; Thermo Fisher Scientific), and rabbit anti–cyclin B2 (1:1,000; PA5-29233; Thermo Fisher Scientific). Secondary Alexa Fluor 680–conjugated (1:10,000; A10043; Thermo Fisher Scientific) or DyLight 800–conjugated (1:10,000; 5257; Cell Signaling Technology) antibodies were used for immunoblotting. Anti–mouse and anti–rabbit Alexa Fluor 680–conjugated light chain–specific secondary antibodies were used (1:5,000) for immunoblotting immunoprecipitations (115–625-174 and 211–622-171; Jackson ImmunoResearch Laboratories, Inc.). Anti–mouse and anti–rabbit Alexa Fluor 488–, 594–, and 647–conjugated secondary antibodies (1:300) were used for immunofluorescence (Thermo Fisher Scientific). CN03 Rho Activator II was from Cytoskeleton. Manganese chloride, thymidine, and RO-3306 were all purchased from Sigma-Aldrich. The CDK1 inhibitor CGP74514A and Wee1 inhibitor MK1775 were from EMD Millipore. CDK2 inhibitor SNS 032 and CDK4/6 inhibitor PD0332991 were from R&D Systems, and roscovitone was from Cell Signaling Technology. The following plasmids used in this study were obtained as gifts: cdc2-HA, cdc2-DN-HA (188818; Addgene; S. van den Heuvel, Utrecht University, Utrecht, Netherlands), R42A cyclin B1–GFP(62) (6184932; Addgene; J. Pines, Institute of Cancer Research, London, England, UK), mCherry-FMNL2 (R. Grosse, University of Marburg, Marburg, Germany), and pLL3.7m-mTurquoise2-SLBP(18–126)-IRES-H1-mMaroon1 (83842; Addgene; M. Lin, Stanford University, Stanford, CA).

### Immunoblotting

Cells were lysed in lysis buffer (150 mM NaCl, 25 mM Tris-HCl, pH 7.4, 1 mM EDTA, 1% [vol/vol] NP-40, 5% [vol/vol] glycerol, 50 µg/ml leupeptin, 50 µg/ml aprotinin, 1 mM 4-(2-aminoethyl)-benzenesulfonyl fluoride, and 1× PhosSTOP phosphatase inhibitor cocktail [Sigma-Aldrich]). Lysates were clarified by centrifugation at 10,000 *g* for 10 min at 4°C.

Cell lysates were separated by SDS-PAGE (4–12% Bis-Tris gels; Thermo Fisher Scientific) under reducing conditions and transferred to nitrocellulose membranes (Whatman). Membranes were blocked for 60 min at RT by using either casein-blocking buffer (Sigma-Aldrich) or 5% (wt/vol) BSA in TBS (10 mM Tris-HCl, pH 7.4, and 150 mM NaCl) containing 0.05% (wt/vol) Tween-20 (TBST) and then probed overnight with primary antibodies diluted in blocking buffer or 5% (wt/vol) BSA/TBST at 4°C. Membranes were washed for 30 min by using TBST and then incubated with the appropriate fluorophore-conjugated secondary antibody diluted in blocking buffer or 5% (wt/vol) BSA/TBST for 45 min at RT in the dark. Membranes were washed for 30 min in the dark by using TBST and then scanned by using the Odyssey infrared imaging system (LI-COR Biosciences), and band intensities were analyzed by using Odyssey software (LI-COR Biosciences). To determine relative amounts of CDK1 in cyclin immunoprecipitations, intensity ratios of CDK1 to cyclin in each cell cycle phase were determined, and then subsequently the fold change in this ratio from S to G2 was calculated.

### Immunoprecipitation

Synchronized cells (two 15-cm-diameter dishes per condition) were lysed (500 µl per dish) in situ at 5 or 9 h after thymidine release as indicated. Lysis was performed at 4°C for 15 min in 150 mM NaCl, 25 mM Tris-HCl, pH 7.4, 1 mM EDTA, 1% (vol/vol) NP-40 (87787; Thermo Fisher Scientific), and protease inhibitors (11836145001; Roche). Lysates were passed five times through a narrow-bore tip before centrifugation (800 *g* for 5 min at 4°C). After centrifugation, immunoprecipitating mAbs (mouse anti–cyclin B1 clone GNS3 or mouse anti–cyclin A clone E67.1; SC53230; Santa Cruz Biotechnology, Inc., or mouse IgG; Sigma-Aldrich) were added to the lysate (2 µg/ml cyclin B1 and 10 µg/ml cyclin A final concentration) together with protein G Sepharose (20 µl of 50% slurry bead volume; GE Healthcare) for 16 h at 4°C. Protein G Sepharose was then collected and washed two times in lysis buffer and once in distilled H_2_O by centrifugation (2,800 *g* for 2 min). Immunoprecipitated complexes were eluted (30 µl) at pH 2 for 5 min at 25°C and neutralized according to the manufacturer’s instructions (88805; Thermo Fisher Scientific). Samples were then reduced at 70°C for 5 min by dilution in 5× sample buffer (125 mM Tris, 10% wt/vol SDS, 25% vol/vol glycerol, 0.01% wt/vol bromophenol blue, and 10% vol/vol β-mercaptoethanol) and subjected to SDS-PAGE and Western blotting by using the Odyssey Infrared Imaging System. To avoid detection of antibody heavy chains, light chain–specific Alexa Fluor 680–conjugated secondary antibodies were used (1:5,000).

### Transfection and viral transduction

HeLa cells were transfected with DNA constructs by using Lipofectamine 2000 reagent (Sigma-Aldrich) and siRNAs by using oligofectamine (Sigma-Aldrich) according to the manufacturer’s instructions. Knockdowns of CDK1, cyclin B1, and cyclin B2 were performed by using SMARTpool reagents (L-003224-00-0005, L-003206-00-0005, and L-003207-00-0005; GE Healthcare) and knockdown of FMNL2 by using a prevalidated Silencer-Select oligonucleotide (human FMNL2; s41620; Thermo Fisher Scientific). The ON-TARGETplus nontargeting siRNA (GE Healthcare) was used as a negative control.

For analysis of asynchronous cells, cells were plated onto glass coverslips for 24 h and then transfected with plasmids and incubated for a further 24 h before analysis. For RNAi-mediated knockdown of proteins, cells were plated for 24 h and then transfected with siRNAs and incubated for a further 72 h before analysis. Rescue of CDK1 knockdown was achieved by coexpressing HA-CDK1 constructs with pEGFP-C1 empty vector for 24 h in siRNA transfected cells. GFP-positive cells were subsequently used for analysis.

For cell cycle analysis, cells were plated for 24 h and then transfected with either plasmid DNA or siRNA for 6 h before a first treatment with thymidine. S phase expression of GFP–cyclin B1–R42A was achieved by synchronizing cells and transfecting the G1 cells immediately after thymidine release for 5 h before fixation. GFP-positive cells were subsequently used for analysis.

For generation of cells stably expressing mTurq2-SLBP_18–126_, lentiviruses were packaged in Lenti-X HEK293T cells (Takara Bio Inc.) by transfection with psPAX2, pMD2.G, and pLL3.7 plasmids by using Lipofectamine 2000. 3 d after transfection, viral supernatant was filtered with a 0.45-µm filter and viral particles concentrated by using PEG-*it* solution. Cells were subsequently transduced with concentrated virus with 8 µg/ml polybrene (Sigma-Aldrich) for 48 h.

### In vitro kinase assay

Purified recombinant GST-tagged cyclin A2–CDK1 and His-tagged cyclin B1–CDK1 were purchased from Invitrogen. 20 ng of each protein and 1 µg substrate GST-tagged FMNL2 C terminus were incubated in 50 mM Hepes, pH 7.4, 150 mM NaCl, 5 mM EDTA, 5 mM DTT, 25 mM MgCl2, 0.02% Triton X-100, and 1 mM ATP at 30°C for 20 min. Kinase reaction was stopped by adding the SDS sample buffer. After running SDS-PAGE and Western blotting, reactions were probed with antiphosphorylated CDK substrate antibody to determine phosphorylation. Samples were also analyzed by MS as described below to identify phosphorylation sites.

### Identification of CDK1 substrates by MS

HeLa cells were plated in 10-cm-diameter dishes, and when 90% confluent, they were treated with either 10 µM RO-3306 (Sigma-Aldrich) or the same volume of DMSO vehicle for 1 h. The medium was removed, the cells were lysed for 30 min in 400 µl lysis buffer (50 mM Tris-HCl, pH 7.4, 150 mM NaCl, 5 mM EDTA, 0.5% (wt/vol) Triton X-100, 50 mM NaF, 1 mM 4-(2-aminoethyl)-benzenesulfonyl fluoride, 1 mM leupeptin, 0.5 µg/ml aprotinin 0.2 mM sodium orthovanadate), and cell debris was removed by centrifugation. Phospho-MAPK/CDK substrates (PXS*P or S*PXR/K) antibody (34B2; Cell Signaling Technology) was added to the supernatant at 1:100 dilution and incubated overnight at 4°C. Immunoprecipitated proteins were isolated by adding 20 µl of 50% slurry of protein G–conjugated Dynabeads (Thermo Fisher Scientific) and incubating for 1 h at RT with rotation. After extensive washing in lysis buffer, proteins were eluted from the beads into reducing SDS-PAGE sample buffer by heating at 70°C for 30 min.

For MS analysis, samples were separated by SDS-PAGE on a 4–12% SDS Bis-Tris gel (Thermo Fisher Scientific), stained for 10 min with Instant Blue (Expedeon), and washed in water overnight at 4°C. Gel pieces were excised and processed by in-gel tryptic digestion as previously described (Horton et al., 2016), and peptides were analyzed by liquid chromatography (LC)-tandem MS as previously described (Robertson et al., 2015). In brief, peptide samples were analyzed by LC-MS by using an UltiMate 3000 Rapid Separation LC system coupled online to an LTQ Velos MS (Thermo Fisher Scientific). Peptides were separated on a bridged ethyl hybrid C18 analytical column (250 mm × 75 µm, 1.7-µm particle size; Waters) by using a 45-min linear gradient from 1 to 25% or 8 to 33% (vol/vol) acetonitrile in 0.1% (vol/vol) formic acid at a flow rate of 200 nl/min. Peptides were selected for fragmentation automatically by data-dependent analysis. MS data were searched by using an in-house Mascot server (version 2.2.03; Matrix Science). Mass tolerances for precursor and fragment ions were 0.4 D and 0.5 D. Data were validated in Scaffold (version 4.4.1.1; Proteome Software) by using a threshold of identification of at least 95% probability at the peptide level, assignment of at least two unique validated peptides, and at least 99% probability at the protein level. These acceptance criteria resulted in an estimated protein false discovery rate of ≤0.1%. Three separate experiments were performed. Relative protein abundance was calculated using the unweighted spectral count of a given protein normalized to the total number of spectra observed in the entire sample. Proteins not identified in all three experiments were removed, and the mean normalized spectral counts for those remaining calculated. Proteins enriched >1.5-fold from control samples versus those treated with CDK1 inhibitor are presented.

### Identification of FMNL2 phosphorylation sites by MS

Two 10-cm-diameter dishes per condition of HeLa cells were plated overnight to 90% confluency, and then each plate was transfected with 5 µg mCherry-FMNL2 by using Lipofectamine 2000 (Sigma-Aldrich) according to manufacturer’s instructions. After 24 h expression, cells were treated with either DMSO or RO-3306 for 1 h and then lysed in lysis buffer (150 mM NaCl, 25 mM Tris-HCl, pH 7.4, 1 mM EDTA, 1% [vol/vol] NP-40, 5% [vol/vol] glycerol, 50 µg/ml leupeptin, 50 µg/ml aprotinin, 1 mM 4-(2-aminoethyl)-benzenesulfonyl fluoride, and 1× PhosSTOP phosphatase inhibitor cocktail; Sigma-Aldrich). Lysates were clarified by centrifugation and then diluted two times with RFP-Trap wash buffer (10 mM Tris-HCl, pH 7.4, 150 mM NaCl, 0.5 mM EDTA). Cherry-FMNL2 was isolated by using 25 µl RFP-Trap Agarose beads (ChromoTek) and incubation at 4°C for 1 h with rotation. After three washes with RFP-Trap wash buffer, proteins were eluted from the beads into reducing SDS-PAGE sample buffer by heating at 70°C for 20 min.

For MS, samples were separated by SDS-PAGE on a 4–12% SDS Bis-Tris gel (Thermo Fisher Scientific), stained for 10 min with Instant Blue (Expedeon), and washed in water overnight at 4°C. Gel pieces were excised and processed by in-gel tryptic digestion as previously described (Horton et al., 2016). Peptides were analyzed by LC-MS/MS by using an UltiMate 3000 Rapid Separation LC (Dionex Corporation) coupled to an Orbitrap Elite MS (Thermo Fisher Scientific). Peptides were separated on a bridged ethyl hybrid C18 analytical column (250 mm × 75 µm internal diameter, 1.7-µm particle size; Waters) over a 45-min gradient from 8 to 33% vol/vol acetonitrile in 0.1% vol/vol formic acid. LC-MS/MS analyses were operated in data-dependent mode to automatically select peptides for fragmentation by collision-induced dissociation. For phosphoproteomic analyses, multistage activation was enabled to fragment product ions resulting from neutral loss of phosphoric acid. Quantification was performed by using Progenesis LC-MS/MS software (Progenesis QI; Nonlinear Dynamics; http://www.nonlinear.com/progenesis/qi-for-proteomics/) as previously described (Horton et al., 2016). In brief, automatic alignment was used, and the resulting aggregate spectrum filtered to include +1, +2, and +3 charge states only. An MGF file representing the aggregate spectrum was exported and searched by using Mascot (one missed cleavage, fixed modification: carbamidomethyl [C]; variable modifications: oxidation [M], phospho [Y], and phospho [ST]; peptide tolerance: ±5 ppm; MS/MS tolerance: ±0.5 D), and the resulting XML file was reimported to assign peptides to features. Four separate experiments were performed, and abundance values for FMNL2 phosphorylated peptides observed in all four experiments were used to determine changes in phosphorylation. Abundance values were normalized within each experiment to the total abundance of FMNL2-assigned peptides and expressed as a ratio relative to DMSO treatment.

### Mutagenesis of FMNL2

Mutagenesis of serine1016 in FMNL2 to alanine or glutamate was performed by overlap extension. A segment of the FMNL2 construct, spanning the site to be mutated from a unique internal HindIII site to a KpnI site at the end of the construct was selected for PCR. The flanking primers complementary to the ends of this target sequence were forward 5′-GAGAGTGACAAGCTTCAAGTCCAG-3′ and reverse 5′-CTTGATGATGGCCATGTTATCCTC-3′, and the two internal primers with complementary ends including the desired mutated codon were forward 5′-GAGCAGCAGGATCCAAAGGCTCCTTCTCATAAATCAAAGAGG-3′ and reverse 5′-CTTCTTTGATTTATGAGAAGGAGCCTTTGGATCCTGCTGCTC-3′ for serine to alanine and forward 5′-GAGCAGCAGGATCCAAAGGAGCCTTCTCATAAATCAAAGAGG-3′ and reverse 5′-CTTCTTTGATTTATGAGAAGGCTCCTTTGGATCCTGCTGCTC-3′ for serine to glutamate. For each PCR reaction, 10 pmol of each primer and 60 ng template was used with 45 µl Platinum PCR supermix (Thermo Fisher Scientific) in a total volume of 50 µl with cycling conditions: 94°C for 2 min followed by 30 cycles of 94°C for 30 s, 55°C for 30 s, and 68°C for 2 min, with a final extension for 5 min at 68°C. The final PCR product was digested with HindIII and KpnI and ligated back into the FMNL2 plasmid cut with the same enzymes. The presence of the desired mutations and integrity of the plasmid were confirmed by sequencing.

### Statistical analysis

Student’s *t* test (unpaired, two tailed, and unequal variance) or ANOVA/Tukey’s multiple comparison test was used to calculate statistical significance as appropriate by using Prism version 7 (GraphPad Software). Statistical significance was given by *, P < 0.05; **, P < 0.01; ***, P < 0.001; and ****, P < 0.0001. All results are displayed as either bar graphs ± SEM or Tukey box and whisker plots (whiskers represent 1.5× interquartile range) and are for at least three biological replicates.

### Online supplemental material

Fig. S1 shows verification of HeLa cell synchronization and cell cycle–dependent changes in adhesion complexes along with images and flow cytometry of G2 cells treated with Mn^2+^ and CN03 Rho activator. Fig. S2 shows inhibition of CDK2 or CDK4/6 does not alter adhesion complex area and also shows images and flow cytometry for CDK1 and cyclin RNAi. Fig. S3 shows changes in FMNL2 phosphorylation during the cell cycle and the effect of expressing FMNL2 phospho mutants on adhesion complex area. Fig. S4 shows accumulation of CDK1–cyclin complexes in the cytosol during G2 and the effect of overexpressing nondegradable cyclin B1 on the adhesion complex area. Video 1 shows live-cell imaging of a HeLa cell expressing GFP-paxillin and mTurq2-SLBP_18–126_ progressing through G2 before rounding up. Video 2 shows live-cell imaging of HeLa cells synchronized by double-thymidine block and then imaged 4 h after release in the presence of vehicle control. Video 3 shows live-cell imaging of HeLa cells synchronized by double-thymidine block and then imaged 4 h after release in the presence of 400 µM Mn^2+^. Video 4 shows live-cell imaging of HeLa cells synchronized by double-thymidine block and then imaged 4 h after release in the presence of 1 mM CN03 Rho activator.

## Supplementary Material

Supplemental Materials (PDF)

Video 1

Video 2

Video 3

Video 4
