# Supplementary material for: Cell adhesion is regulated by CDK1 during the cell cycle
Source: J Cell Biol. 2018 Sep 3;217(9):3203–18. doi: 10.1083/jcb.201802088 (PMC6122981; doi:10.1083/jcb.201802088)
Supplement: Supplemental Materials (PDF) [file JCB_201802088_sm.pdf]

Supplemental material

Jones et al., https://doi.org/10.1083/jcb.201802088

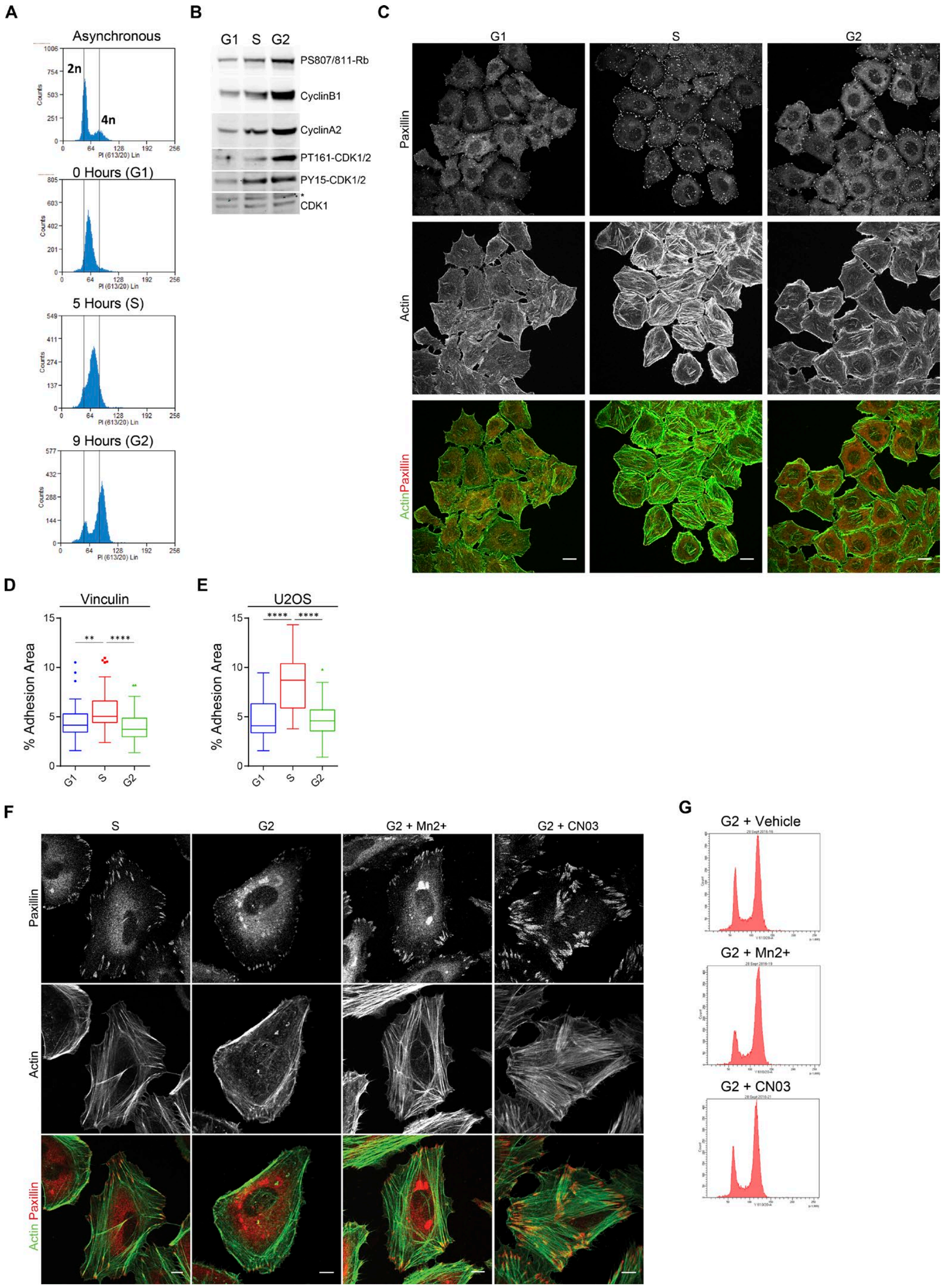

Figure S1. **Verification of cell cycle progression in double-thymidine block synchronized HeLa cells and cell cycle–dependent changes in adhesion complexes. (A)** Flow cytometric analysis of synchronized HeLa cells released for 0, 5, and 9 h then fixed and stained with propidium iodide. **(B)** Western blot analysis of cell cycle markers after HeLa synchronization and release. **(C)** Immunofluorescence images of multiple synchronized cells in G1, S, and G2 phase stained for adhesion marker paxillin and actin. Bars, 20 µm. **(D)** Quantification of adhesion complex area changes across the cell cycle in HeLa cells stained for the adhesion marker vinculin. A minimum of 42 cells per condition was used for analysis. **(E)** Quantification of paxillin-stained adhesion complex area changes across the cell cycle in synchronized U2OS cells. A minimum of 27 cells per condition was used for analysis. **(F)** Images of cells in S phase or in G2 phase treated with vehicle, Mn^{2+}, or CN03. Bars, 10 µm. **(G)** Flow cytometric analysis of synchronized HeLa cells released for 9 h in the presence of vehicle, Mn^{2+}, or CN03 and then fixed and stained with propidium iodide. Results in D and E are displayed as Tukey box and whisker plots (whiskers represent 1.5× interquartile range) and are for at least three biological replicates. **, P < 0.01; ****, P < 0.0001.

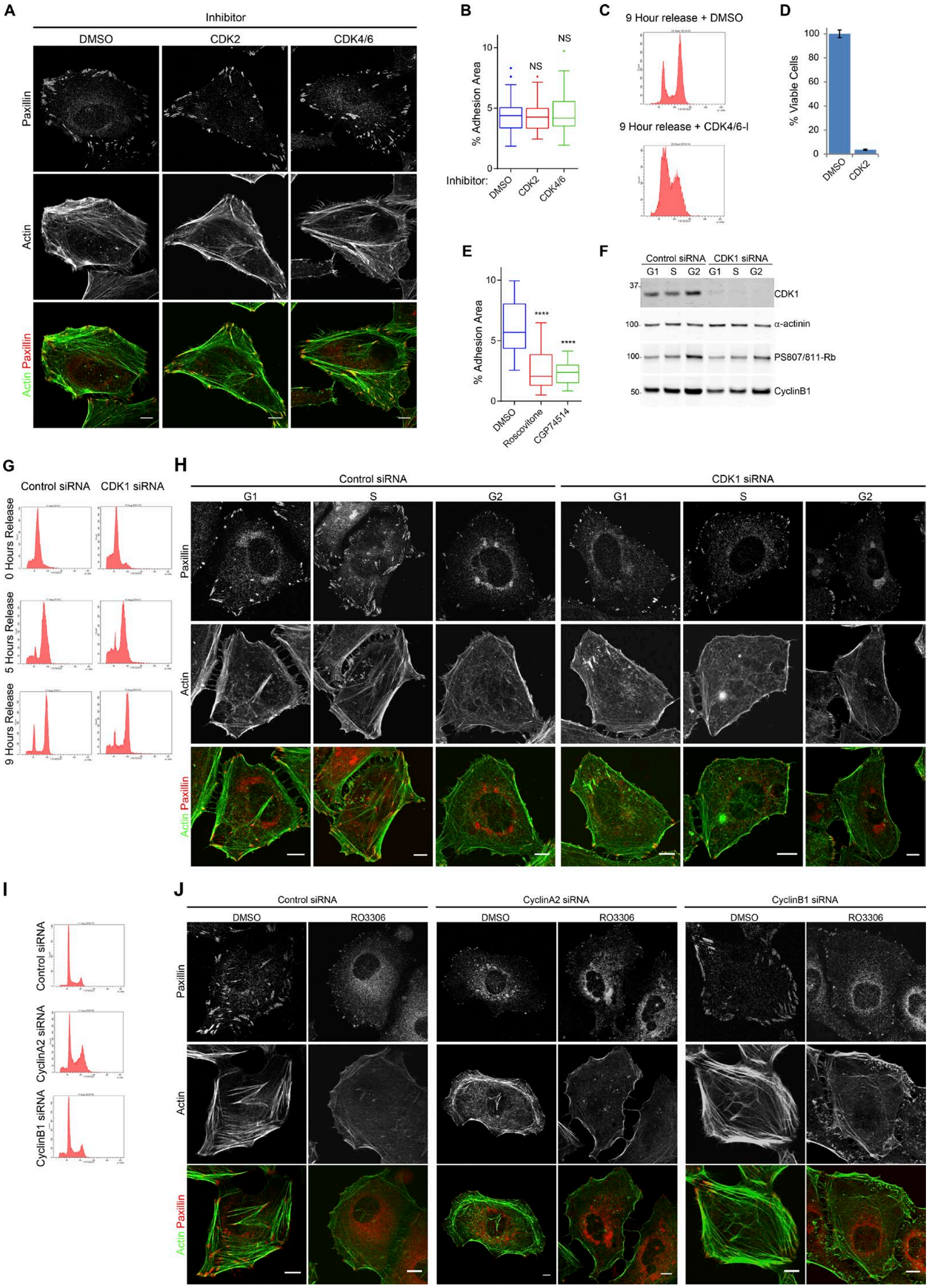

Figure S2. **Selectivity of the effect of inhibition of 1 on adhesion complexes and verification of CDK knockdown and cyclin A2 expression is required for the effect of inhibition of CDK1 on adhesion complexes. (A)** Immunofluorescence images of cells plated on glass coverslips for 48 h and then treated with either DMSO, CDK2 inhibitor SNS-032, or CDK4/6 inhibitor PD0332991 and stained for paxillin and actin. **(B)** Quantification of adhesion complex area per cell of DMSO-, SNS-032-, or PD0332991-treated cells. A minimum of 33 cells per condition was used for analysis. **(C)** Flow cytometry analysis of synchronized HeLa cells released for 9 h in the presence of DMSO or CDK4/6 inhibitor PD0332991. **(D)** Quantification of cell viability by using WST-1 proliferation reagent of cells treated for 48 h with either DMSO or CDK2 inhibitor SNS-032. **(E)** Quantification of adhesion complex area per cell of DMSO and the CDK1 inhibitors roscovitone- or CGP74514A-treated cells. A minimum of 33 cells per condition was used for analysis. **(F)** Western blot showing knockdown of CDK1 in synchronized HeLa cells. **(G)** Flow cytometric analysis of synchronized control or CDK1-knockdown cells released for 0, 5, or 9 h and then fixed and stained with propidium iodide. Molecular masses are given in kilodaltons. **(H)** Images of control or CDK1-knockdown cells in G1, S, or G2 phase stained for paxillin and actin. **(I)** Flow cytometric analysis of propidium iodide–stained asynchronous control, cyclin A2–, or cyclin B1–knockdown HeLa cells. **(J)** Immunofluorescence images of control, cyclin A2–, or cyclin B1–knockdown cells plated on glass coverslips and then treated with either DMSO or CDK1 inhibitor RO3306 and stained for paxillin and actin. Bars, 10 µm. Results in D are displayed as bar graphs ± SEM and in B and E as Tukey box and whisker plots (whiskers represent 1.5× interquartile range) and are for at least three biological replicates. ****, $P < 0.0001$.

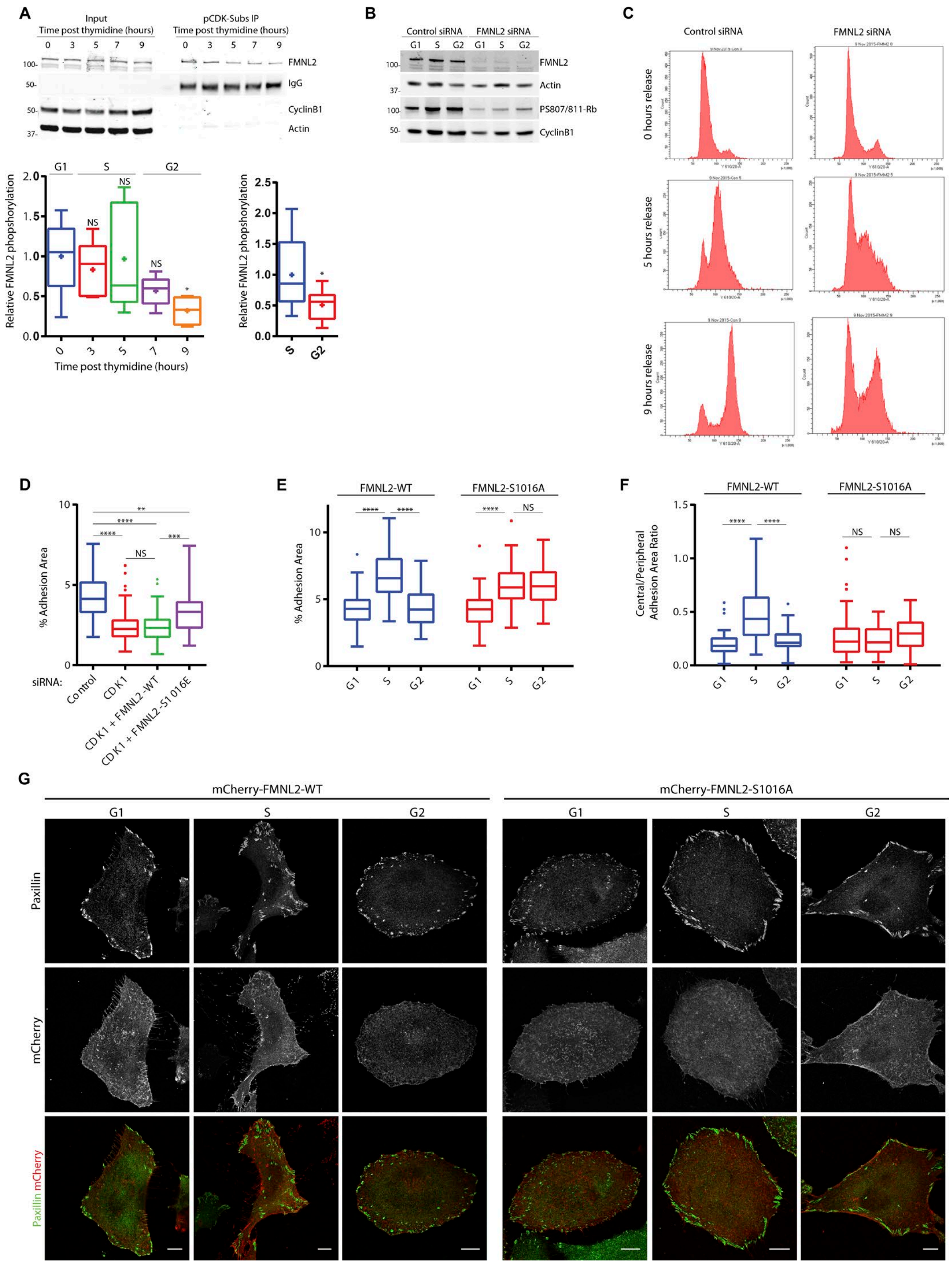

Figure S3. **Expression of S1016A-FMNL2 or knockdown of FMNL2 prevents adhesion complex disassembly in G2. (A)** Immunoprecipitation (IP) of anti-CDK/MAPK substrate antibody and Western blot of FMNL2 from HeLa cells arrested by double-thymidine block and then released for the time periods indicated with quantification of FMNL2 phosphorylation relative to time 0 from five independent experiments presented. For analysis of FMNL2 phosphorylation in S and G2, mean band intensities for 3- and 5-h release along with 7- and 9-h release were compared. **(B)** Western blot showing knockdown of FMNL2 in synchronized HeLa cells. Molecular masses are given in kilodaltons. **(C)** Flow cytometric analysis of synchronized control or FMNL2-knockdown cells released for 0, 5, or 9 h and then fixed and stained with propidium iodide. **(D)** Quantification of adhesion complex area per cell after CDK1 knockdown and expression of WT-FMNL2 or S1016E-FMNL2. A minimum of 47 cells per condition was used for analysis. **(E)** Quantification of adhesion complex area changes in G1, S, and G2 phase for WT-FMNL2– and S1016A-FMNL2–expressing cells. A minimum of 46 cells per condition was used for analysis. **(F)** Quantification of ratio of central adhesion complex area (adhesion complex area >3 µm from cell periphery) to peripheral adhesion complex area (adhesion complex area <3 µm from cell periphery) in G1, S, and G2 phase for WT-FMNL2– and S1016A-FMNL2–expressing cells. A minimum of 47 cells per condition was used for analysis. **(G)** Immunofluorescence images of WT-FMNL2– or S1016A-FMNL2–expressing cells in G1, S, or G2 phase stained for paxillin and actin. Bars, 10 µm. Results in A and D–F are displayed as Tukey box and whisker plots (whiskers represent 1.5× interquartile range) and are for at least three biological replicates. *, $P < 0.05$; **, $P < 0.01$; ***, $P < 0.001$; ****, $P < 0.0001$.

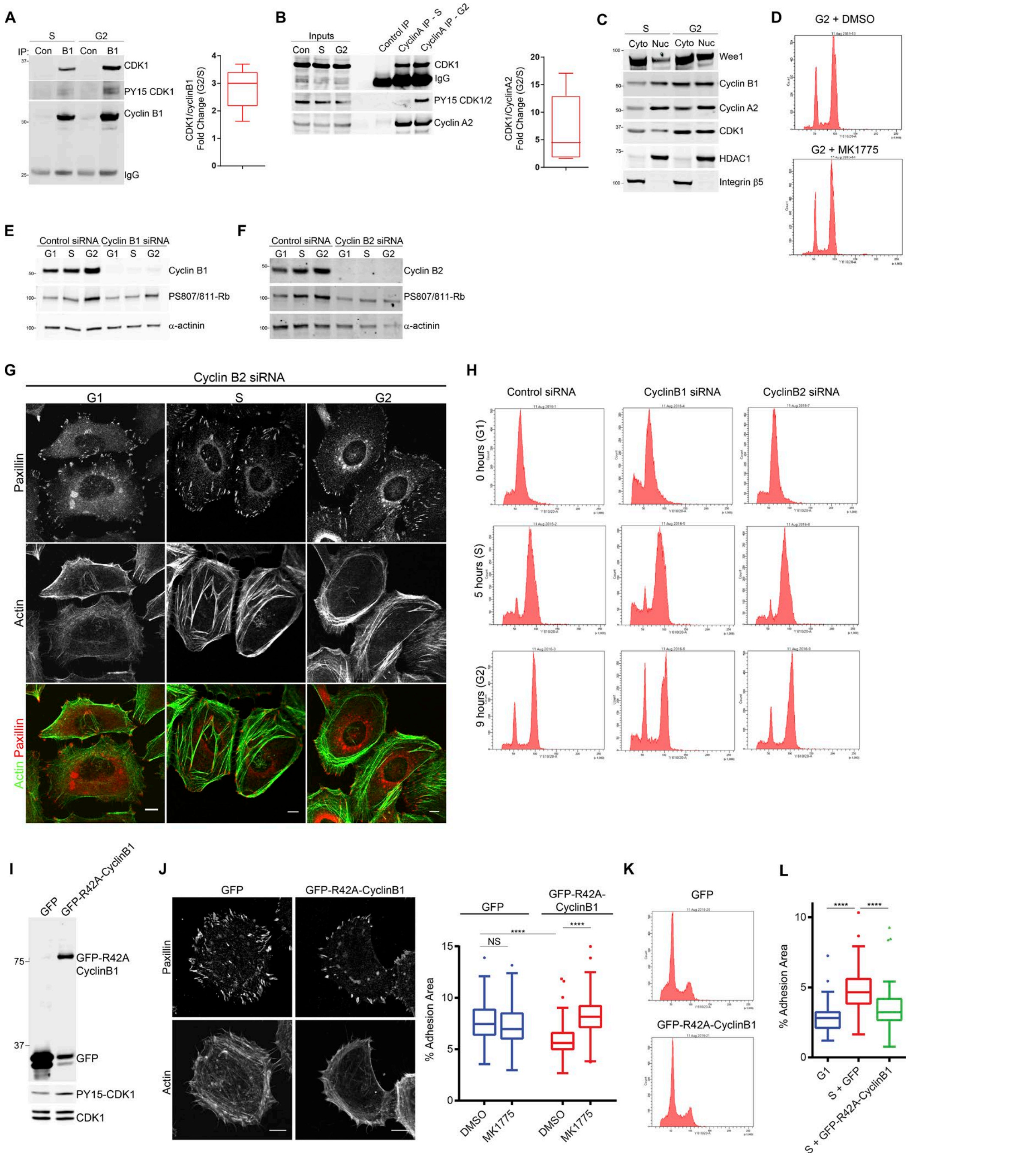

Figure S4. **Expression of cyclin B1 and inhibition of CDK1 by Wee1 is required for adhesion complex disassembly in G2 phase. (A)** Left: Immunoprecipitation (IP) of cyclin B1 and Western blot showing increased levels of CDK1 associated with cyclin B1 in G2 phase compared with S phase. Right: Quantification of fold increase in CDK1 associated with cyclin B1 in G2 phase relative to S phase from five separate immunoprecipitations. **(B)** Left: Immunoprecipitation of cyclin A and Western blot showing increased levels of CDK1 associated with cyclin A in G2 phase compared with S phase. Right: Quantification of fold increase in CDK1 associated with cyclin A in G2 phase relative to S phase from six separate immunoprecipitations. **(C)** Western blot of nuclear (Nuc) and cytoplasmic (Cyto) cellular fractions from S and G2 phase cells. **(D)** Flow cytometric analysis of synchronized cells released for 7 h, then treated for 2 h with either DMSO or MK1775, and then fixed and stained with propidium iodide. **(E)** Western blot showing knockdown of cyclin B1 in synchronized HeLa cells. **(F)** Western blot showing knockdown of cyclin B2 in synchronized HeLa cells. **(G)** Immunofluorescence images of cyclin B2–knockdown cells in G1, S, or G2phase stained for paxillin and actin. Bars, 10 µm. **(H)** Flow cytometric analysis of synchronized control, cyclin B1–, or cyclin B2–knockdown cells released for 0, 5, or 9 h and then fixed and stained with propidium iodide. **(I)** Western blot showing overexpression of GFP–cyclin B1–R42A in asynchronous cells. Molecular masses are given in kilodaltons. **(J)** Left: Immunofluorescence images of cells expressing either GFP or GFP–R42A–cyclin B1 stained for paxillin and actin. Right: Quantification of adhesion complexes in cells expressing GFP or overexpressing nondegradable cyclin B1–R42A treated with DMSO or MK1775 for 2 h. A minimum of 74 cells per condition was used for analysis. **(K)** Flow cytometric analysis of asynchronous cells expressing GFP or GFP-R42A–cyclin B1 for 48 h and then fixed and stained with propidium iodide. **(L)** Quantification of adhesion area per cell of synchronized cells transiently overexpressing nondegradable cyclin B1–R42A in S phase. A minimum of 56 cells per condition was used for analysis. Bars, 10 µm. Results in A, B, J, and L are displayed as Tukey box and whisker plots (whiskers represent 1.5× interquartile range) and are for at least three biological replicates. ****, P < 0.0001.

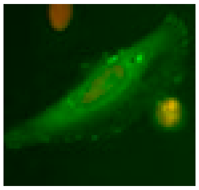

Video 1. **Live-cell imaging of a HeLa cell expressing GFP-paxillin (adhesion marker, green) and mTurq2-SLBP$_{18–126}$ (nuclear fucci marker, pseudocolored red) progressing through G2 before rounding up.** Loss of nuclear mTurq2-SLBP$_{18–126}$ signal begins at the end of S phase and therefore marks progression of cell cycle into G2. Images were taken every 10 min, and the playback speed is seven frames/s.

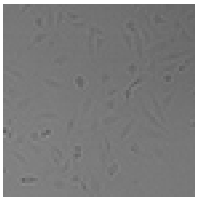

Video 2. **Live-cell imaging of HeLa cells synchronized by double-thymidine block and then imaged 4 h after release in the presence of vehicle control.** Images were taken every 10 min for an additional 12 h, and the playback speed is seven frames/s.

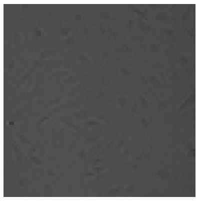

Video 3. **Live-cell imaging of HeLa cells synchronized by double-thymidine block and then imaged 4 h after release in the presence of 400 µM Mn^{2+}.** Images were taken every 10 min for an additional 12 h, and the playback speed is seven frames/s.

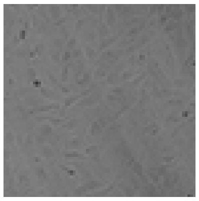

Video 4. **Live-cell imaging of HeLa cells synchronized by double-thymidine block and then imaged 4 h after release in the presence of 1 mM CN03 Rho activator.** Images were taken every 10 min for an additional 12 h, and the playback speed is seven frames/s.
